# Effects of maturity stage and lactic acid bacteria on the fermentation quality and aerobic stability of Siberian wildrye silage

**DOI:** 10.1002/fsn3.312

**Published:** 2016-06-21

**Authors:** Ping Li, Shiqie Bai, Minghong You, Yixin Shen

**Affiliations:** ^1^ Sichuan Academy of Grassland ScienceChengduChina; ^2^ College of Grasland ScienceNanjing Agricultural UniversityNanjingChina

**Keywords:** Fermentation quality, lactic acid bacteria, Siberian wildrye silage, Tibetan Plateau

## Abstract

It is difficult to make good quality of silage from alpine gramineous from the Qinghai Tibetan plateau. The effects of lactic acid bacteria (LAB) on the fermentation quality and aerobic stability of Siberian wildrye silage were studied in southeast of the Qinghai Tibetan plateau. Siberian wildrye materials were freshly cut at the sprouting stage, flowering stage, and milky stage. Silage was prepared by using a small‐scale silage fermentation system (bag silos). *Lactobacillus plantarum* (LP, 5 × 10^8^ cfu/kg FM), *Lactobacillus buchneri* (LB, 5 × 10^8^ cfu/kg FM) and their mixture (LP+LB, 5 × 10^8^ cfu/kg FM) as silage additives were separately added to ensiled forages, and no additive served as control (CK). These bag silos were kept at room temperature (<15°C), and the silage qualities were analyzed after 60 days of ensiling. The number of indigenous LAB on fresh materials was less than that of yeasts and molds, and LAB species showed specification adapted to low temperature. LAB inoculated silages had lower (*P *<* *0.05) pH value, NH_3_‐N/TN and butyric acid content compared with control silage. Silage treated with LB had higher contents of acetic acid, propionic acid, WSC and CP. However, the aerobic stability of silages inoculated with LAB did not differ significantly between stages (*P *>* *0.05). When fermentation characteristics, chemical composition, and aerobic stability were considered, treatment with *L. plantarum* resulted in high quality of Siberian wildrye silage harvested at the flowering stage in the alpine region.

## Introduction

Siberian wildrye (*Elymus sibiricus* L.), as the type species of the genus Elymus, is a perennial, self‐pollinating and allotetraploid grass indigenous to Northern Asia (Dewey 1974). Its geographic distribution extends from Sweden to Japan and even to parts of Alaska and Canada (Bowden and Cody 1961), and then extends southerly to Tibetan Plateau (Ma et al. 2008). In subalpine meadows with less than 4000 m altitude in the Qinghai Tibetan plateau, Siberian wildrye usually serves as an important feed resource for ruminants, because of its several desirable agronomic traits such as high yield, hardness, disease tolerance, and high nutrient values, which is comparable to that of oat and highland barley. However, the growing season of this forage is very short (from May to August), and the harvesting time is during rainy season, resulting in feed shortages. Because Siberian wildrye harvest is seasonal, it is normally ensiled to provide a continuous supply for ruminants such as yak and Tibetan sheep.

It is difficult to make good quality of silage from alpine gramineous (*i.e*., oat and reed canary grass), because of low temperature, insufficient lactic acid bacteria (LAB), and intensive UV radiation in Qinghai Tibetan plateau (Li et al. 2012). To improve the fermentation quality and decrease nutrient loss during ensiling, LAB is applied to promote the adequate lactic acid production, decrease pH in silage and improve safe feeding of the livestock. However, the application efficiency of LAB was affected by fermentable substrate, temperature, and so on. *Lactobacillus plantarum* was shown to quickly produce lactic acid, but it could not improve the aerobic stability of grass silages due to the low acetic acid content in silage. *Lactobacillus buchneri* could increase aerobic stability of silage, but the DM loss would increase for the production of high acetic acid and propionic acid (Weinberg et al. 2010; Randby et al. 2012). Therefore, we propose, regardless of maturity stage of ensiling materials, application of LAB could improve silage quality in alpine regions, like as in median or tropical regions.

Most grass silage information regarding fermentation quality and aerobic stability has been obtained from other crops, to our knowledge, and limited information was available on Siberian wildrye silage in the alpine region. The objective of this study was to investigate the effects of treatments with *L. plantarum*,* L. buchneri*, and the combination of both on the fermentation quality and aerobic stability of Siberian wildrye silage.

## Materials and Methods

### Treatments

Two experiments were conducted in the experiment base of the Sichuan Academy of Grassland Science, which was located on the southeast edge of the Tibetan Plateau (N 31°51′‐33°33′, E 101°51′‐103°22′). Siberian wildrye seeds were sown in early April, mid‐April, and early May. In early July, the ensiling materials were fresh crops with different growing stages (heading stage, flowering stage, and milky stage), and chopped with a laboratory‐type chopper to about 2–3 cm, ensiled in repeated polyethylene bags (25 × 30 cm), and vacuumized. Each polyethylene bag was filled with about 1 kg (fresh weight) of chopped forages, then stored at ambient temperature (<15°C). Fresh and ensiled forages (three polyethylene bags per treatment) were sampled for analysis of microbial composition, fermentation quality, and chemical composition after 60 days of ensiling. At the end of the ensiling period, the silages were subjected to an aerobic stability test. The following treatments were applied:

1. Control (CK, no additives, 5 mL/kg FM (fresh matter) 0.9% physiological saline).
2. *Lactobacillus plantarum* (LP, the main component was *Lactobacillus plantarum*, produced by Medipharm USA). The stated total number of microorganisms was 2.0 × 10^9^ cfu/g powder.
3. *Lactobacillus buchneri* (LB, the main component was *Lactobacillus buchneri*, produced by Medipharm USA). The stated total number of micro‐organisms was 7.6 × 10^11^ cfu/g powder.
4. Combination of *Lactobacillus plantarum* and *Lactobacillus buchneri* (LP+LB, the main components were natural lactic acid with partial enzymes and minerals, which was produced by Microferm Ltd. UK). The state total number of microorganisms was 6.66 × 10^11^ cfu/g powder.

The inoculants were applied as follows: on the day of the experiment, each inoculant was diluted to about 1.0 × 10^8^ cfu/ml by 0.9% physiological saline. The diluted inoculants was sprayed on the ensiling materials 5 ml/kg FM.

### Analytical methods

Chemical analysis was performed in triplicate. The dry matter (DM) content of the fresh materials and silages after deactivation of enzymes were determined by oven drying for 48 h at 65°C. Crude protein (CP) was determined by the Kjeldahl method (AOAC, 1990). Neutral detergent fiber (aNDF) and acid detergent fiber (ADF) contents were analyzed with a modified procedure, using sulphite and amylase (Van Soest et al. 1991). The water‐soluble carbohydrate (WSC) content was determined by the anthrone reaction‐rate assay (Murphy 1958). NH_3_‐N was determined in the silages by the extraction of 20 g of frozen samples with 180 mL distilled water for 3 min in a Stomacher blender (Weatherburn 1967). The silage juice for the measurement of pH was made, using a portable pH meter. The lactic acid (LA), acetic acid (AA), propionic acid (PA), and butyric acid (BA) levels were determined by high performance liquid chromatography (HPLC) (Muck and Dickerson 1988).

A portion of the water extracts was used for the enumeration of LAB by pour plating on Man Rogosa Sharpe agar (MRS), and incubated at 30°C for 48–72 h. The yeasts and molds were determined by pour plating serial 10‐fold dilutions of the eater extracts on malt extract agar (Oxoid CM0059; Thermo Fisher Scientific Inc., Waltham, MA), which had been acidified with lactic acid (concentration of 850 g/kg and added at 50 g/kg, vol/vol). The plates were incubated at 32°C for 48–72 h, and those containing a minimum of 30 and a maximum of 300 colony‐forming units were enumerated (Reich and Kung 2010). The single colony was selected for further 16S r RNA identification (the work was completed by College of Life Science, Sichuan University).

The remaining silages with some treatments were mixed and placed (without packing) into clean silos, and a thermocouple wire was inserted into the center of the silage mass. The thermocouple wires were connected to a data logger (model number CR10X; Campbell Scientific, Inc., Logan, UT), which recorded the temperature every 10 min, then averaged the temperature over a 30‐min period. Aerobic stability was defined as the time it took for the temperature in the silage masses to rise to 2°C above ambient temperature.

### Statistical analysis

The factorial analysis of variance was carried out using the procedure of SAS 8.2 (SAS Institute Inc., Cary, NC) to determine the effects of the treatments. The statistical model used was as follows: *Y*_*ijk*_ = *μ* + *α*_*i*_ + b_*j*_ + (*α* × b)_*ij*_ + e_*ijk*,_ where *Y*_*ijk*_ is the fermentation characteristics, chemical composition, and aerobic stability of ensiled forages; *μ* is the overall mean; *α*_*i*_ is the effect of forage maturity stage i (i = 1, 2, 3); (*α* × *b*)_*ij*_ is the effect of interaction between forage maturity stage and additive treatment; and e_*ijk*_ is the error term. Contracts of means were constructed to evaluate the effects of treatments within the maturity stages, using the Tukey's test, and significance was declared at *P *<* *0.05. The parameters of the chemical composition profiles and fermentation profiles of the silages treated with LAB at different maturity stages were plotted when LAB × maturity stages were significant, to aid the interpretation of the results.

## Results

### Chemical composition and microbial analysis of fresh crops

The chemical composition and microbial analysis for fresh crops are shown in Table 1. The number of LAB was far less than those of the yeast and molds (*P *<* *0.05). As the stage prolonged, the CP content and number of LAB decreased, but the concentration of aNDF and ADF, and the number of yeasts and molds all increased. There were 13 species identified by 16S rRNA, and the species of LAB included *Lactobacillus planttarum*,* Lactobacillus spentosus*,* Lactobacillus casei*,* Lactobacillus brevis*,* Lactobacillus fermentum*,* Enterococcus faeculis*,* Enterococcus durans*,* Pediococus acidilactiolsp*,* Straphylococcus delphini*,* Straphylococcus epidermindis*,* Pedicococus acidilactici*,* Brochothrix campestris*, and *Weissella confusa*.

**Table 1:** Chemical and microbiological analysis of ensiling materials

Stage	%FM	% DM				log Cfu/g DM		
	DM	WSC	CP	NDF	ADF	LAB	Yeast	Molds
Heading	30.38^c^	7.57	12.26^a^	53.90^c^	32.68^c^	2.3^ab^	4.7^c^	4.1^b^
Flowering	34.83^b^	7.13	7.01^c^	59.39^a^	38.47^a^	2.8^a^	6.1^a^	5.9^a^
Milky	40.85^a^	7.14	9.55^b^	56.17^ab^	38.62^a^	1.9^b^	6.3^a^	5.8^a^
Standard error of mean (SEM)	0.48	0.08	0.23	0.35	0.27	0.04	0.07	0.05

Means of DM content at ensiling within column with different superscript letters differ (*P *<* *0.05).

### Fermentation quality and aerobic stability of Siberian wildrye silage

LAB and forage maturity stage had effects on the fermentation profiles of silages (Table 2). The silages treated with LAB had lower (*P *<* *0.05) pH and BA content. LAB increased LA content, and LB increased (*P *<* *0.05) volatile fatty acid content, compared with control silage. As the plant aged, the pH value increased, the concentrations of LA and AA decreased (*P *<* *0.05), and no significant (*P *>* *0.05) difference between maturity stages was found about AA, PA, and aerobic stability. LAB had significant (*P *<* *0.05) effects on the fermentation characteristics and aerobic stability of silage, but maturity stage had little (*P *>* *0.05) effect on BA content in silage.

**Table 2:** The effect of LAB and maturity stage on the fermentation quality and aerobic stability of silage

Treatment	Maturity stage	pH	%DM				%TN	Aerobic stability (h)
			LA	AA	PA	BA	NH3‐N	
No additive (CK)	Sprouting stage	4.35^b^	1.69^g^	0.52^e^	0.22^g^	0.14^b^	10.30^a^	98.67^gh^
	Flowering stage	4.40^ab^	2.38^e^	0.92^cd^	0.16^ghi^	0.17^ab^	5.96^d^	92.00^h^
	Milky stage	4.52^a^	1.70^g^	0.90^cd^	0.32^f^	0.19^a^	7.38^c^	107.33^g^
*Lactobacillus plantrum* (LP)	Sprouting stage	3.75^f^	3.86^a^	0.82^d^	0.15^hi^	0.06^c^	8.60^b^	156.67^f^
	Flowering stage	4.13^e^	2.78^d^	0.82^d^	0.12^i^	0.04^cde^	6.25^d^	191.33^d^
	Milky stage	4.31^bc^	2.69^d^	0.38^f^	0.17^gh^	0.06^c^	5.55^d^	155.00^f^
*Lactobacillus buchneri* (LB)	Sprouting stage	3.82^f^	3.10^c^	1.61^a^	0.82^b^	0.01^de^	7.09^c^	241.00^a^
	Flowering stage	4.28^bcd^	2.38^e^	1.48^b^	0.75^c^	0.05^c^	9.07^b^	224.33^b^
	Milky stage	4.20^cde^	2.22^ef^	0.93^cd^	0.98^a^	0.01^de^	6.12^d^	206.67^c^
LP+LB	Sprouting stage	4.14^de^	3.32^b^	0.96^c^	0.52^d^	0.03^cde^	9.07^b^	171.67^e^
	Flowering stage	4.34^bc^	2.78^d^	0.21^g^	0.49^d^	0.01^e^	7.71^c^	200.67^cd^
	Milky stage	4.32^bc^	2.13^f^	0.80^f^	0.43^e^	0.04^cd^	5.96^d^	170.00^e^
Standard error of mean (SEM)		0.04	0.10	0.07	0.05	0.01	0.26	7.96
Treatment		<0.001	<0.001	<0.001	<0.001	<0.001	0.001	<0.001
Maturity stage		<0.001	<0.001	<0.001	<0.001	0.120	<0.001	<0.001
Treatment × Maturity stage		<0.001	<0.001	<0.001	<0.001	0.008	<0.001	<0.001

Means of treatment within column with different superscript letters differ (*P *<* *0.05).

### Chemical composition of Siberian wildrye silage

The effects of LAB on the chemical composition of Siberian wildrye silage are shown in Table 3. Except of DM and aNDF, LAB showed significant (*P *<* *0.05) effects on the chemical composition of the silage. Silage treated with LB had higher concentrations of CP and aNDF. The silages treated with LP or LB had higher (*P *<* *0.05) concentrations of WSC and ADF.

**Table 3:** The effect of LAB and maturity stage on chemical composition of silage

Treatment	Maturity stage	%FM	%DM			
		DM	WSC	CP	aNDF	ADF
No additive (CK)	Sprouting stage	27.69^g^	0.85^cde^	11.17^b^	56.69^b^	34.28^ef^
	Flowering stage	34.21^d^	0.85^cde^	7.49^cde^	62.25^a^	40.48^bc^
	Milky stage	41.54^a^	1.13^b^	5.52^fg^	61.41^a^	41.39^b^
*Lactobacillus plantrum* (LP)	Sprouting stage	29.48^f^	0.90^cd^	12.77^a^	55.49^b^	33.65^f^
	Flowering stage	34.43^d^	0.60^g^	8.08^cd^	62.87^a^	44.38^a^
	Milky stage	39.13^b^	0.68^efg^	6.47^ef^	63.51^a^	44.52^a^
*Lactobacillus buchneri* (LB)	Sprouting stage	31.22^e^	1.06^bc^	12.28^ab^	55.47^b^	35.61^de^
	Flowering stage	35.37^d^	1.21^ab^	7.52^cde^	60.84^a^	44.22^a^
	Milky stage	36.87^c^	0.63^fg^	6.88^de^	61.45^a^	43.90^a^
LP+LB	Sprouting stage	27.47^g^	1.41^a^	10.98^b^	56.79^b^	36.07^d^
	Flowering stage	34.50^d^	0.61^g^	8.60^c^	61.48^a^	38.80^c^
	Milky stage	42.15^a^	1.19^ab^	4.74^g^	60.36^a^	39.27^c^
Standard error of mean (SEM)		0.81	0.05	0.44	0.54	0.67
Treatment		0.824	<0.001	0.012	0.444	<0.001
Maturity stage		<0.001	<0.001	<0.001	<0.001	<0.001
Treatment × Maturity stage		<0.001	<0.001	0.031	0.575	<0.001

Means of treatment within column with different superscript letters differ (*P *<* *0.05).

## Discussion

### Effects of chemical and microbiological composition of fresh forage on the quality of silage

Both LAB and WSC are the most important factors affecting fermentation quality and nutritive value of silage. Although there occurred 13 kinds of LAB species, the number of indigenous LAB on the surface of Siberian wildrye was lower than that of yeasts and molds (Table 1), which was in accordance to our previous study (Li et al. 2012). In the present experiment, maturity stage also affected the microbial counts with numbers of yeast, mold, and indigenous LAB decreasing with increasing maturity at harvest, which was inconsistent with results of Muller (2009) who reported that later harvest stages resulted in increased yeast, mold and LAB counts in the preconserved herbage. The propagation and metabolic activity of LAB species is intensively related to environment. For example, *Weissella confus* detected in silage could grow and propagate in the alpine region with the characteristics of low temperature resistance, high acid resistance, and utilization of a wide variety of carbon sources during ensiling (Flaherty et al. 2003; Yang et al. 2014). The WSC content of fresh forage at different maturity stage was >6.0–7.0% DM recommended theoretical requirement to achieve well preserved fermentation of typical gramineae forage (Smith 1962; Teller et al. 2012). Silage without any inoculant had higher pH value (4.35–4.52) and butyric acid content (0.14–0.19% DM) (Table 2), resulting in bad silage quality. Thus, it was LAB rather than WSC to determine the quality of Siberian wildrye silage in the alpine pastoral of the Tibetan Plateau.

### Effects of maturity stage and LAB on fermentation quality of silage

It was necessary to select appropriate LAB to improve fermentation quality of silage in alpine pastoral region. Borreani et al. (2009) showed that *L. platarum* will improve the fermentation quality at appropriate DM contents (30–35%, but this value may vary depending on the plants). In our study, the addition of *L. plantarum* increased concentrations of LA, AA and PA (*P *<* *0.05), decreased pH value and concentrations of NH_3_‐N and BA in silages (*P *<* *0.05), compared with control silage (Table 2). Occurrence of higher acetic acid in control silage and silage treated with *L. plantarum* indicated fresh forage occupied more hetero‐fermentation LAB species such as *L. brevis*,* L. fermentum*, (the numbers were not shown in Table 1). Recently, more and more attention has been given to *L. buchneri* (Santos et al. 2013). Unfortunately, *L. buchneri* are not good candidates for grass silage inoculation, and may even worsen the quality of the silage fermentation by increasing DM losses and production of acetic acid throughout the process. *L. buchneri* produced more NH_3_‐N than *L. plantarum* (*P *>* *0.05) in silage, which was in agreement with the study of Hu et al. (2009) and Contreras‐Govea et al. (2013). Although the silages inoculated by *L. buchneri* alone or in combine with *L. plantarum* showed lower pH and higher acetic acid content (Table 2), it preferred to applied *L. plantarum* because of lower DM loss and higher CP content (Table 3) in the alpine region.

### Effects of maturity stage and LAB on chemical composition of silage

The effects of LAB on Chemical composition of silage varied, to some extent, by maturity stage of ensiling materials. The increase in DM content at ensiling with increasing maturity make Siberian wildrye progressively more difficulty to ensile as pointed out by Buxton and O'Kiely (2003) for many different crops. In present study, the CP, aNDF, and ADF concentrations of the silage ensiled at sprouting stage differed (*P *<* *0.05) from those ensiled at the flowering and milky stages, which may have been caused by the fact that accumulation of digestible organic matter ceases after the flowering stage for providing energy for reproductive growth (Khan et al. 2012). When the silage preparing occurred at the sprouting stage and flowering stage, the silage treated with LP owned more CP, comparing with LB‐treated silage. This is inconsistent with previous studies which reported that silages treated with LB would consume more nutrients to produce acetic acid, resulting in lower WSC and CP in silage (Kleinschmit and Kung 2006; Schimidt et al. 2009). Comparing with the single treatment, treatment LP+LB increased WSC in silage at the sprouting stage and milk stage, which may be due to higher number of *L.plantarum* in the ensling materials. Thus, it was the best harvesting time at the flowering stage of the Siberian wildrye in the alpine region.

### Effects of maturity stage and LAB on aerobic stability of silage

Aerobic stability is an important trait of silages as it determines the safety and quality of the preserved forage upon exposure to air. In present study, the resistant to deterioration (>72 h) of Siberian wildrye silages treated with or without LAB was comparable with that of other forages (Abdelhadi and Tricarico 2009; Weinberg et al. 2010; Arriola et al. 2011, 2012). The major subjective of the current study was to explore the main effects of maturity stage, inoculation, and their interactions on the aerobic stability of Siberian wildrye silage. The factorial analysis (Table 2) revealed maturity stage effect on aerobic stability, which tended to increase firstly, and then decrease as the plant aged. May be the higher level of acetic acid and propionic acid in silages of flowering stage restrained development of aerobic fungi and yeast, and were associated with higher aerobic loss. Meanwhile, the lower aerobic stability of silages at sprouting stage and milk stage resulted from either higher level of residual WSC or lower level of fermentation products. From results in Table 2, *L. buchneri* and *L. plantarum* were assumed to be involved in the aerobic stability of the Siberian wildrye silage. Previous studies illustrated the inoculation of homo‐fermentative LAB has been shown to facilitate yeast growth and deterioration on exposure to air (Driehuis et al. 2001; Nishino and Touno 2005). *L. plantarum* was reported to be unsuccessful in suppressing aerobic spoilage when inoculated alone (Filya 2003; Hu et al. 2009). In the present study, *L. plantarum* strains did not enhance the aerobic deterioration of grass silages, which was consistent with the study of Chen and Weinberg (2009) who reported that lower temperature inhibits the fungi to consume water soluble carbohydrate and lactic acid for propagation. Hetero‐fermentive LAB species produced acetic acid and propionic acid, which have the potential to avoid aerobic deterioration. Because of advantages of *L. buchneri* over *L. plantarum* as in our study, the resistance of Siberan wildrye silage to deterioration could be due to activity of *L. buchneri*. The acetic acid content of 0.52–0.90 g/kg DM accounts for higher aerobic stability of the control silage. Wang and Nishino (2013) illustrated that spoilage should not occur after long‐term storage regardless of the storage temperature or if the silage was used in a region where the climatic conditions were similar to the silage‐producing factory regardless of the storage period. It is believed that the aerobic stability of silage at low temperature is necessary to discuss the activity and function of yeast.

## Conclusions

It confirmed that the number of LAB was less than that of yeast and molds, and varied between maturity stage, the species of LAB occurred to specification adapted to low temperature. Addition of LAB was effective in improving the fermentation quality and aerobic stability. It also influenced the chemical compositions. These inoculants have the potential to improve the gramineous forage fermentation quality in the alpine region. When the fermentation quality, chemical composition, and aerobic stability were considered, the single treatment with *L. plantarum* at the flowering stage resulted in high quality of Siberian wildrey silage in the alpine pastoral region of the Qinghai Tibetan plateau.

## Conflict of Interest

None declared.
